# Assessment of Liver Enzymes and the Risk of Non-alcoholic Fatty Liver Disease (NAFLD) Among Information Technology (IT) Professionals

**DOI:** 10.7759/cureus.88786

**Published:** 2025-07-25

**Authors:** Thirumoorthi Natarajan, Shenbaga Lalitha S, Sujatha Lakshminarayanan, Balakrishnan Madhavan, Devi Krishna Ravichandran, Krishna Prasanth

**Affiliations:** 1 Department of Surgical Gastroenterology, Cancer Institute (Women's India Association; WIA), Chennai, IND; 2 Department of Biochemistry, Tagore Medical College &amp; Hospital, Chennai, IND; 3 Department of Ophthalmology, Sree Balaji Medical College and Hospital, Chennai, IND; 4 Department of General Medicine, Kauvery Hospital, Chennai, IND; 5 Department of Community Medicine, Sree Balaji Medical College and Hospital, Chennai, IND

**Keywords:** alt, ast, ggt, it professionals, liver enzymes, metabolic health, nafld, occupational wellbeing, public health, sedentary lifestyle

## Abstract

Background: Non-alcoholic fatty liver disease (NAFLD) is a growing public health concern, particularly among individuals with sedentary occupations. Liver enzyme alterations are often early indicators of hepatic steatosis. The information technology (IT) workforce, due to prolonged screen time and limited physical activity, may be particularly vulnerable to NAFLD.

Objective: To assess the prevalence of NAFLD and liver enzyme abnormalities (alanine aminotransferase (ALT), aspartate aminotransferase (AST), and gamma-glutamyl transferase (GGT)) among IT professionals and to explore associations with lifestyle and metabolic parameters.

Methods: This cross-sectional observational study included 250 IT professionals aged between 25 and 55 years from a tertiary care setting. Data were collected using structured questionnaires, anthropometric assessments, liver function tests (LFTs), and abdominal ultrasonography. Multivariate logistic regression was used to identify independent predictors of NAFLD.

Results: The mean age of participants was 36.4 ± 6.8 years, with 172 males (68.8%) and 78 females (31.2%). NAFLD was identified in 92 participants (36.8%) via ultrasonography. Elevated ALT levels were observed in 78 participants (31.2%), elevated AST in 52 participants (20.8%), and elevated GGT in 61 participants (24.4%). Among those with NAFLD (n=92), mean BMI was 29.1 kg/m² compared to 26.1 kg/m² in those without NAFLD (p<0.001). Similarly, mean ALT levels were higher in the NAFLD group (50.2 U/L vs. 36.1 U/L, p<0.001). Multivariate analysis identified BMI ≥27 kg/m² (n=143, 57.2%), elevated ALT (n=78, 31.2%), elevated GGT (n=61, 24.4%), and sedentary lifestyle (n=161, 64.4%) as independent predictors of NAFLD.

Conclusion: The findings of this study underscore a significant presence of NAFLD and liver enzyme elevations among individuals working in sedentary professional environments, such as the IT sector. The observed associations with modifiable lifestyle factors, particularly physical inactivity and elevated body mass, suggest a pressing need for routine metabolic and hepatic health assessments in such occupational groups. Early identification, coupled with targeted lifestyle modification strategies, holds the potential to mitigate the long-term health risks associated with NAFLD and improve overall workforce well-being.

## Introduction

Non-alcoholic fatty liver disease (NAFLD) has emerged as one of the most prevalent chronic liver disorders globally, affecting approximately 25% of the adult population [1]. NAFLD encompasses a clinical continuum, ranging from simple hepatic steatosis to non-alcoholic steatohepatitis (NASH), which may progress to fibrosis, cirrhosis, and hepatocellular carcinoma if undiagnosed or left untreated [2,3]. The rising prevalence of NAFLD parallels the global surge in obesity and type 2 diabetes mellitus (T2DM), particularly in urban populations experiencing sedentary lifestyles and poor metabolic health [4,5].

While NAFLD is often considered a hepatic manifestation of metabolic syndrome, it is also increasingly observed in individuals without overt obesity or diabetes, indicating the multifactorial and lifestyle-linked nature of the disease [6]. Among these factors, insulin resistance plays a pivotal role in hepatic lipid accumulation and metabolic dysregulation [7].

Serum aminotransferases, namely alanine aminotransferase (ALT), aspartate aminotransferase (AST), and gamma-glutamyl transferase (GGT), are commonly used as non-invasive markers of hepatic injury in NAFLD. Elevated levels of ALT and GGT, in particular, are often observed in affected individuals and may serve as useful screening tools despite limitations in predicting histologic severity [8,9].

Emerging research has begun to explore the occupational and environmental determinants of NAFLD. Sedentary occupations, such as those in the information technology (IT) sector, are linked to prolonged screen time, low physical activity, high stress, and irregular dietary habits, all of which may promote visceral adiposity and insulin resistance [10,11]. However, there is limited epidemiological data on the prevalence and risk factors of NAFLD within specific occupational groups like IT professionals in India.

This study was conducted to estimate the prevalence of NAFLD and liver enzyme abnormalities (ALT, AST, GGT) in a sample of IT professionals and to identify key sociodemographic, behavioral, and metabolic predictors associated with the disease. Findings from this study aim to inform early preventive measures and screening policies within high-risk occupational settings.

## Materials and methods

Study design and setting

This cross-sectional observational study was conducted to assess liver enzyme profiles and the prevalence of NAFLD among employees in the IT sector. The study was carried out between May 2024 and March 2025 at Sree Balaji Medical College and Hospital, Chennai, India, in collaboration with some IT companies in the region.

Sample size and sampling technique

A total of 250 full-time IT professionals were recruited using stratified random sampling to ensure balanced representation across various occupational roles, including software development, technical support, and operations. The sample size was calculated to detect a minimum clinically significant difference of 10 U/L in ALT levels, assuming a standard deviation of 25, with 80% power and a 95% confidence level. To compensate for potential dropouts or incomplete data, a recruitment target of 275 was initially set.

Eligibility criteria

Eligible participants were full-time IT professionals aged between 25 and 55 years, with a minimum of one year of experience in a desk-based role. Individuals with a history of alcohol consumption exceeding 20 grams per day were excluded, as were those with a known diagnosis of chronic liver disease, such as viral hepatitis or autoimmune hepatitis. Additional exclusion criteria included the use of hepatotoxic medications, pregnancy, and incomplete clinical or biochemical data. All participants provided written informed consent prior to enrollment.

Ethical considerations

The study protocol was reviewed and approved by the Institutional Human Ethics Committee of Sree Balaji Medical College and Hospital, Bharath Institute of Higher Education and Research, Chennai, under reference number 505/SBMCH/IHEC/2024/2153, dated May 5, 2024. The research adhered to the ethical principles outlined in the Declaration of Helsinki and the Indian Council of Medical Research (ICMR) guidelines for biomedical research involving human participants.

Data collection

Participants were evaluated during a scheduled single-day assessment session. A structured questionnaire was administered to collect demographic information (age and gender), work-related characteristics (daily working hours and screen exposure), and lifestyle variables (physical activity levels, dietary habits, sleep duration, and tobacco use). Anthropometric parameters, including height, weight, waist circumference, and blood pressure, were measured using standardized methods. Body mass index (BMI) was calculated as weight in kilograms divided by the square of height in meters.

Following an overnight fast of eight to 10 hours, venous blood samples were collected for biochemical analysis. The parameters assessed included liver enzymes ALT, AST, GGT, and alkaline phosphatase (ALP), as well as other liver function indicators such as serum bilirubin, total protein, and albumin. In addition, fasting blood glucose and lipid profiles were evaluated to assess metabolic status.

All participants underwent abdominal ultrasonography, performed by a certified radiologist using standardized imaging criteria. Hepatic steatosis was diagnosed based on increased echogenicity of the liver parenchyma relative to the renal cortex, posterior beam attenuation, and blurring of vascular margins.

Diagnostic criteria for NAFLD

A diagnosis of NAFLD was established when ultrasonographic evidence of hepatic steatosis was present in the absence of significant alcohol intake or other secondary causes of fat accumulation in the liver. Liver enzyme elevation was defined based on laboratory reference values: ALT greater than 40 U/L, AST greater than 35 U/L, and GGT levels exceeding 50 U/L in males and 30 U/L in females.

Statistical analysis

Data were analyzed using IBM SPSS Statistics for Windows, Version 26.0 (IBM Corp., Armonk, NY, USA). Descriptive statistics were computed for all variables. Continuous variables were presented as means with standard deviations, while categorical variables were reported as absolute frequencies and percentages.

Comparative analyses between groups with elevated versus normal liver enzymes were performed using the independent sample t-test or the Mann-Whitney U test, based on the normality of data distribution. Pearson or Spearman correlation coefficients were used to assess relationships between liver enzymes and variables such as BMI, physical activity, and NAFLD status. A multivariate logistic regression model was constructed to identify independent predictors of NAFLD, adjusting for relevant confounders. A p-value of less than 0.05 was considered statistically significant.

## Results

Demographic and clinical characteristics

A total of 250 IT professionals were enrolled and analyzed. The mean age was 36.4 ± 6.8 years. The cohort consisted of 172 males (68.8%) and 78 females (31.2%). The mean BMI was 27.3 ± 3.8 kg/m², with 179 participants (71.6%) classified as overweight or obese (BMI ≥ 25 kg/m²). A sedentary lifestyle, defined as engaging in physical activity for less than 150 minutes per week, was reported by 161 participants (64.4%). The mean daily screen time was 8.9 ± 1.7 hours. The demographic parameters are given below in Table 1. 

**Table 1:** Demographic and clinical profile of the study participants (n = 250)

Parameter	Mean ± SD or n (%)
Age (years)	36.4 ± 6.8
Male	172 (68.8%)
Female	78 (31.2%)
BMI (kg/m²)	27.3 ± 3.8
Overweight/Obese (BMI ≥ 25 kg/m²)	179 (71.6%)
Daily screen time (hours)	8.9 ± 1.7
Sedentary lifestyle (<150 min/week)	161 (64.4%)

Liver enzyme and biochemical profile

Biochemical analysis revealed elevated ALT levels (>40 U/L) in 78 participants (31.2%), AST levels (>35 U/L) in 52 participants (20.8%), and GGT levels above sex-specific cut-offs in 61 participants (24.4%), as shown below in Table 2. The mean ALT, AST, and GGT levels were 41.8 ± 16.7 U/L, 34.2 ± 11.4 U/L, and 47.5 ± 19.3 U/L, respectively. All participants had values for ALP, bilirubin, albumin, fasting glucose, and lipid profile measured as part of the liver function and metabolic panel; however, no significant trends were observed in those parameters across NAFLD categories and thus are not tabulated here.

**Table 2:** Liver enzyme levels and abnormality prevalence (n = 250)

Liver enzyme	Mean ± SD (U/L)	Elevated n (%)	Reference range (U/L)
Alanine aminotransferase (ALT)	41.8 ± 16.7	78 (31.2%)	7 – 40
Aspartate aminotransferase (AST)	34.2 ± 11.4	52 (20.8%)	10 – 40
Gamma-glutamyl transferase (GGT)	47.5 ± 19.3	61 (24.4%)	8 – 40

Prevalence of NAFLD

Ultrasonographic examination identified hepatic steatosis consistent with NAFLD in 92 participants (36.8%). Among them, 55 (59.8%) had Grade I steatosis, 32 (34.8%) had Grade II, and 5 (5.4%) had Grade III. Participants with NAFLD were more likely to have elevated BMI, liver enzymes, and a sedentary lifestyle, as shown in Table 3.

**Table 3:** Comparison of participants with and without non-alcoholic fatty liver disease (NAFLD)

Variable	NAFLD (n = 92)	No NAFLD (n = 158)	p-value	Reference range
BMI (kg/m²)	29.1 ± 3.6	26.1 ± 3.4	<0.001	18.5 – 24.9
Alanine aminotransferase (ALT) (U/L)	50.2 ± 15.4	36.1 ± 12.8	<0.001	7 – 40
Gamma-glutamyl transferase (GGT) (U/L)	56.8 ± 17.9	41.3 ± 15.2	<0.001	8 – 40
Sedentary lifestyle n (%)	68 (73.9%)	92 (58.2%)	0.02	<150 min activity/week

A significantly greater proportion of individuals with NAFLD had elevated ALT (63.0% vs. 17.7%) and elevated GGT (57.6% vs. 13.3%) compared to those without NAFLD (p < 0.001 for both). Although AST was elevated in some participants, it did not show a significant association with NAFLD status in subgroup analysis (p > 0.05).

Multivariate logistic regression

Multivariate logistic regression analysis was performed to identify independent predictors of NAFLD as given in Table 4. The final model included BMI, ALT, GGT, AST, and sedentary behavior. The analysis demonstrated that BMI ≥ 27 kg/m², ALT > 40 U/L, elevated GGT, and sedentary lifestyle were statistically significant predictors of NAFLD. AST did not retain statistical significance in the adjusted model.

**Table 4:** Multivariate logistic regression analysis for predictors of non-alcoholic fatty liver disease (NAFLD)

Variable	Odds ratio (OR)	95% confidence Interval	p-value	Reference threshold
BMI ≥ 27 kg/m²	3.4	1.8 – 6.2	<0.001	Normal: <25 kg/m²
Alanine aminotransferase (ALT) > 40 U/L	2.9	1.5 – 5.4	0.001	Normal: 7 – 40 U/L
Gamma-glutamyl transferase (GGT) above normal range	2.2	1.1 – 4.1	0.02	Normal: 8 – 40 U/L
Sedentary lifestyle	1.8	1.02 – 3.2	0.04	<150 min activity/week

The results highlight a high prevalence of NAFLD and liver enzyme abnormalities among IT professionals. Obesity, elevated ALT and GGT levels, and sedentary behavior were significantly associated with hepatic steatosis. These findings reinforce the need for targeted screening and early lifestyle interventions in occupational health settings.

## Discussion

This cross-sectional study examined the prevalence of NAFLD and liver enzyme abnormalities among IT professionals, a group increasingly vulnerable to metabolic diseases due to sedentary work habits, prolonged screen time, and irregular lifestyle patterns. Among 250 participants, 92 (36.8%) had NAFLD, while 78 (31.2%) showed elevated ALT levels. These findings reflect the growing metabolic risk in this occupational group and underscore the utility of liver enzymes as early noninvasive markers of hepatic dysfunction.

NAFLD and lifestyle risk factors

The prevalence of NAFLD observed in our cohort aligns with findings from other sedentary and metabolically vulnerable populations. In a recent study, Kim et al. reported that body fat distribution was significantly associated with NAFLD and fibrosis risk across racial and ethnic groups, emphasizing adiposity as a key driver of hepatic steatosis [12]. Similarly, Atri et al. identified an NAFLD prevalence of 73.6% among morbidly obese South Indian women undergoing bariatric surgery, further underscoring the role of obesity in disease development [13]. In our study, both elevated BMI and insufficient physical activity emerged as independent predictors of NAFLD, echoing the associations described by Zawdie et al. in an Ethiopian population-based analysis [14].

Liver enzyme abnormalities were consistently elevated among participants with NAFLD. Elevated levels of ALT and GGT were significantly associated with ultrasound-confirmed steatosis in our cohort. These findings mirror those of Mandal et al., who demonstrated that ALT and GGT are strongly correlated with hepatic fat accumulation and insulin resistance in NAFLD patients with T2DM [15]. However, although AST levels were elevated in some cases, they did not retain statistical significance in multivariate analysis, supporting the conclusions of Lai et al., who emphasized that AST is less specific than ALT for detecting early hepatic steatosis [16].

While gender differences in NAFLD risk and liver enzyme expression have been reported in broader population studies, our analysis did not reveal significant variation in NAFLD prevalence or liver enzyme abnormalities between male and female participants. Although males in our cohort exhibited marginally higher mean ALT and GGT levels, these differences were not statistically significant. This may be due to sample size limitations or occupational homogeneity within the cohort. Future studies with larger and more gender-balanced populations may help clarify the role of sex-based physiological or behavioral factors in occupational NAFLD risk.

Limitations of enzyme markers alone

Despite the clinical value of ALT and GGT as screening tools, exclusive reliance on these enzymes may overlook significant hepatic pathology. In an early study, Guha et al. demonstrated that individuals with normal transaminase levels could still exhibit substantial hepatic steatosis or fibrosis when evaluated using imaging modalities or elastography, highlighting the limitations of enzyme-based screening alone [17]. The pathophysiological basis for such findings is further elucidated by Isaza et al., who explored how occupational exposures like intermittent hypoxia contribute to oxidative liver damage [18]. Similarly, Sundaram et al. observed that nocturnal hypoxia-induced oxidative stress, particularly in individuals with disordered sleep patterns, may accelerate NAFLD progression independently of liver enzyme abnormalities [19].

These insights underscore the importance of integrating liver biochemistry with anthropometric assessments and lifestyle profiling. A multifactorial approach can enhance diagnostic accuracy and allow for earlier identification of at-risk individuals who might otherwise be missed by enzyme thresholds alone.

Occupational vulnerability

The information technology professionals in our study reported an average daily screen time of 8.9 hours, and 161 participants (64.4%) did not meet the World Health Organization’s recommended physical activity levels. This occupational profile mirrors findings by Song et al., who observed a strong association between long working hours, reduced activity, and NAFLD risk in Korean office workers [20]. The interplay of chronic stress, prolonged sedentary behavior, and erratic meal patterns likely fosters visceral adiposity and hepatic lipid accumulation, which are core components in the pathogenesis of NAFLD. These findings underscore the critical need for structured workplace wellness programs focused on physical activity, nutrition, and stress management.

At a molecular level, Lebensztejn et al. have highlighted the role of hepatokines, hormone-like substances secreted by the liver, in modulating systemic metabolism [21]. In individuals with NAFLD, dysregulated hepatokine profiles contribute to insulin resistance and chronic low-grade inflammation, thereby reinforcing the bidirectional link between hepatic and metabolic dysfunction.

Broader metabolic context

Though individuals with diagnosed diabetes were excluded, the elevated liver enzyme patterns and metabolic indicators observed in our cohort are comparable to those documented in prediabetic populations. Nguyen et al. emphasized that NAFLD often represents the hepatic facet of metabolic syndrome, with elevated transaminases serving as early biochemical markers of glycemic dysregulation [22]. Building on this, Chalasani et al. outlined in their clinical guidance that broader metabolic screening, including glucose tolerance and insulin resistance assessments, should be routinely performed in patients suspected of having NAFLD [23].

To further enhance diagnostic precision, advanced tools such as transient elastography and non-invasive fibrosis scoring indices (e.g., Fibrosis-4 Index (FIB-4)) have been recommended. Wong et al. highlighted their utility in detecting early fibrosis and guiding follow-up in at-risk populations [24]. While these were beyond the scope of the current cross-sectional design, they represent important considerations for future workplace screening protocols and longitudinal follow-up strategies.

Implications for intervention

Lifestyle modification remains the primary and most effective approach for managing NAFLD. Interventions that promote physical activity, caloric moderation, and weight reduction have consistently demonstrated histological improvement, as seen in both adults and children. This has been substantiated by Romero-Gómez et al. [25] in adult cohorts and by Nobili et al. [26] in pediatric populations. Building on this, Adams and Angulo [27] and Eslam et al. [28] advocate for redefining NAFLD as metabolic dysfunction-associated fatty liver disease (MAFLD), highlighting its intrinsic metabolic roots rather than its exclusionary diagnostic criteria.

Beyond lifestyle changes, pharmacological innovations are gaining traction. Loomba et al. [29] have demonstrated that semaglutide, a GLP-1 receptor agonist, significantly improves NASH outcomes in individuals with concurrent metabolic syndrome. Meanwhile, adjunctive treatments like phosphatidylcholine, as explored by Maev et al. [30], have shown efficacy in improving liver enzymes and broader metabolic profiles, supporting a comprehensive, multi-modal treatment strategy.

Strengths and limitations of the study

A major strength of this study is its targeted focus on a high-risk occupational cohort, IT professionals, whose sedentary work environment and lifestyle make them particularly vulnerable to metabolic and hepatic dysfunction. The study employed standardized ultrasound criteria along with comprehensive biochemical profiling, which enhanced diagnostic accuracy for NAFLD.

The geographical setting of Chennai, one of India’s largest and most prominent IT hubs in India, adds contextual relevance and generalizability to the findings. The study population included individuals not only from urban areas but also from neighboring states such as Kerala and Andhra Pradesh, as well as rural parts of Tamil Nadu. This heterogeneity reflects a broad cross-section of dietary habits and lifestyle patterns seen across urban Indian IT populations.

Additionally, although gender was included as a variable in our initial exploratory analyses, it did not emerge as a significant moderator of NAFLD or liver enzyme patterns. However, we acknowledge that sex-specific differences in NAFLD pathophysiology exist and that our study may have been underpowered to detect such variations. Future research with larger and more diverse samples may provide more clarity on gender-specific risk profiles in occupational settings.

However, the cross-sectional design restricts causal inference, and the absence of advanced fibrosis assessment tools such as FibroScan or liver biopsy limits staging precision. Additionally, while lifestyle parameters such as physical activity were assessed, other important contributors such as dietary quality, sleep duration, and psychosocial stress were not formally measured. These factors could confound or mediate the observed associations with NAFLD. For instance, irregular eating habits and poor sleep quality, both common in IT settings, are known to promote insulin resistance, visceral adiposity, and hepatic lipid deposition. Future multicentric and longitudinal studies should incorporate structured assessments using validated dietary recall tools, sleep quality indices, and stress inventories to provide a more comprehensive understanding of lifestyle determinants in this population.

## Conclusions

This study demonstrates a notable burden of NAFLD and abnormal liver enzyme profiles among IT professionals, reflecting the growing metabolic risks linked to sedentary occupational environments. The association of NAFLD with elevated liver enzymes, increased BMI, and physical inactivity highlights the silent progression of liver dysfunction in asymptomatic working populations.

These findings underscore the importance of early screening and preventive health strategies within workplace settings. Incorporating routine liver function testing and abdominal ultrasonography into employee wellness programs may facilitate the timely detection of NAFLD and support lifestyle-based interventions aimed at mitigating long-term hepatic and metabolic complications.

## References

[1] Younossi ZM, Koenig AB, Abdelatif D, Fazel Y, Henry L, Wymer M (2016). Global epidemiology of nonalcoholic fatty liver disease-meta-analytic assessment of prevalence, incidence, and outcomes. Hepatology.

[2] Pappachan JM, Babu S, Krishnan B, Ravindran NC (2017). Non-alcoholic fatty liver disease: a clinical update. J Clin Transl Hepatol.

[3] Han SK, Baik SK, Kim MY (2023). Non-alcoholic fatty liver disease: definition and subtypes. Clin Mol Hepatol.

[4] Jennison E, Patel J, Scorletti E, Byrne CD (2019). Diagnosis and management of non-alcoholic fatty liver disease. Postgrad Med J.

[5] Memaj P, Jornayvaz FR (2022). Non-alcoholic fatty liver disease in type 1 diabetes: prevalence and pathophysiology. Front Endocrinol (Lausanne).

[6] Praveenraj P, Gomes RM, Kumar S (2015). Prevalence and predictors of non-alcoholic fatty liver disease in morbidly obese South Indian patients undergoing bariatric surgery. Obes Surg.

[7] Sheng X, Che H, Ji Q (2018). The relationship between liver enzymes and insulin resistance in type 2 diabetes patients with nonalcoholic fatty liver disease. Horm Metab Res.

[8] Xuan Y, Wu D, Zhang Q, Yu Z, Yu J, Zhou D (2024). Elevated ALT/AST ratio as a marker for NAFLD risk and severity: insights from a cross-sectional analysis in the United States. Front Endocrinol (Lausanne).

[9] Ulasoglu C, Enc FY, Kaya E, Yilmaz Y (2019). Characterization of patients with biopsy-proven non-alcoholic fatty liver disease and normal aminotransferase levels. J Gastrointestin Liver Dis.

[10] Kaul A, Bansal N, Sharma P, Aneja S, Mahato MP (2023). Association of screen time usage and physical activity with overweight and obesity among school-going children in Uttar Pradesh. Cureus.

[11] Edwardson CL, Gorely T, Davies MJ (2012). Association of sedentary behaviour with metabolic syndrome: a meta-analysis. PLoS One.

[12] Kim D, Cholankeril G, Ahmed A (2024). Association between body fat distribution and nonalcoholic fatty liver disease/fibrosis based on race/ethnicity. J Obes Metab Syndr.

[13] Atri A, Jiwanmall SA, Nandyal MB (2020). The prevalence and predictors of non-alcoholic fatty liver disease in morbidly obese women - a cross-sectional study from southern India. Eur Endocrinol.

[14] Zawdie B, Tadesse S, Wolide AD, Nigatu TA, Bobasa EM (2018). Non-alcoholic fatty liver disease and associated factors among type 2 diabetic patients in southwest Ethiopia. Ethiop J Health Sci.

[15] Mandal A, Bhattarai B, Kafle P (2018). Elevated liver enzymes in patients with type 2 diabetes mellitus and non-alcoholic fatty liver disease. Cureus.

[16] Lai X, Chen H, Dong X (2024). AST to ALT ratio as a prospective risk predictor for liver cirrhosis in patients with chronic HBV infection. Eur J Gastroenterol Hepatol.

[17] Guha IN, Parkes J, Roderick PR, Harris S, Rosenberg WM (2006). Non-invasive markers associated with liver fibrosis in non-alcoholic fatty liver disease. Gut.

[18] Isaza SC, Del Pozo-Maroto E, Domínguez-Alcón L, Elbouayadi L, González-Rodríguez Á, García-Monzón C (2020). Hypoxia and non-alcoholic fatty liver disease. Front Med (Lausanne).

[19] Sundaram SS, Halbower A, Pan Z (2016). Nocturnal hypoxia-induced oxidative stress promotes progression of pediatric non-alcoholic fatty liver disease. J Hepatol.

[20] Song E, Kim JA, Roh E (2021). Long working hours and risk of nonalcoholic fatty liver disease: Korea National Health and Nutrition Examination Survey VII. Front Endocrinol (Lausanne).

[21] Lebensztejn DM, Flisiak-Jackiewicz M, Białokoz-Kalinowska I, Bobrus-Chociej A, Kowalska I (2016). Hepatokines and non-alcoholic fatty liver disease. Acta Biochim Pol.

[22] Nguyen QM, Srinivasan SR, Xu JH, Chen W, Hassig S, Rice J, Berenson GS (2011). Elevated liver function enzymes are related to the development of prediabetes and type 2 diabetes in younger adults: the Bogalusa Heart Study. Diabetes Care.

[23] Chalasani N, Younossi Z, Lavine JE (2018). The diagnosis and management of nonalcoholic fatty liver disease: practice guidance from the American Association for the Study of Liver Diseases. Hepatology.

[24] Wong VW, Adams LA, de Lédinghen V, Wong GL, Sookoian S (2018). Noninvasive biomarkers in NAFLD and NASH - current progress and future promise. Nat Rev Gastroenterol Hepatol.

[25] Romero-Gómez M, Zelber-Sagi S, Trenell M (2017). Treatment of NAFLD with diet, physical activity and exercise. J Hepatol.

[26] Nobili V, Marcellini M, Devito R (2006). NAFLD in children: a prospective clinical-pathological study and effect of lifestyle advice. Hepatology.

[27] Adams LA, Angulo P (2006). Treatment of non-alcoholic fatty liver disease. Postgrad Med J.

[28] Eslam M, Sanyal AJ, George J (2020). MAFLD: A consensus-driven proposed nomenclature for metabolic associated fatty liver disease. Gastroenterology.

[29] Newsome PN, Buchholtz K, Cusi K (2021). A placebo-controlled trial of subcutaneous semaglutide in nonalcoholic steatohepatitis. N Engl J Med.

[30] Maev IV, Samsonov AA, Palgova LK, Pavlov CS, Shirokova EN, Vovk EI, Starostin KM (2020). Effectiveness of phosphatidylcholine as adjunctive therapy in improving liver function tests in patients with non-alcoholic fatty liver disease and metabolic comorbidities: real-life observational study from Russia. BMJ Open Gastroenterol.

